# Can a novel lysophosphatidic acid receptor 1 antagonist ACT in time to halt pulmonary fibrosis and improve breathing?

**DOI:** 10.1016/j.jpet.2025.103610

**Published:** 2025-06-05

**Authors:** Bradley T. Andresen

**Affiliations:** Department of Biotechnology and Pharmaceutical Sciences, College of Pharmacy, Western University of Health Sciences, Pomona, California

**Keywords:** Idiopathic pulmonary fibrosis, lysophosphatidic acid, lysophosphatidic acid receptor 1

Idiopathic pulmonary fibrosis (IPF) is a debilitating disease with poor treatment options. IPF has an incidence rate between 0.09 and 1.30 per 10,000 people and an estimated prevalence of 0.33–4.51 per 10,000 people (Maher et al, 2021). Current predictions of the world population are above 8.2 billion people; thus, between 73,800 and over a million people are diagnosed with IPF each year, and 270,600 to over 3.5 million people are stricken with IPF worldwide. Although many patients worldwide likely do not have access to treatments, current treatments with the pan-tyrosine kinase inhibitor nintedanib and the antifibrotic and antioxidant agent pirfenidone only slow the progression of IPF. Untreated IPF has a median mortality of 3 years (Cottin et al, 2021). Treatment of IPF with nintedanib and pirfenidone slowed down the rate of lung function decline after 1 year of treatment, but only pirfenidone decreased deaths within the first year of treatment (King et al, 2014; Richeldi et al, 2014). Thus, novel treatments are necessary to control IPF. In a recently published article in *The Journal of Pharmacology and Experimental Therapeutics*, Birker-Robaczewska et al (2025) propose a tantalizing novel therapy for IPF, noncompetitive inhibition of lysophosphatidic acid (LPA) receptor 1.

The etiology of IPF is complex and involves multiple profibrotic ligands and the upregulation of receptors, including LPA1 (Kadam and Schnitzer, 2024). In addition to the increase in LPA1 expression, LPA levels increase in patients with IPF, and greater LPA levels are statistically associated with increased risk for exacerbations and respiratory hospitalization (Neighbors et al, 2023). Additionally, LPA signaling through LPA1 increases pulmonary fibrosis (Tager et al, 2008) and through LPA2 leads to transforming growth factor (TGF)-*β* activation through an integrin-dependent pathway (Xu et al, 2009). TGF-*β* independently leads to increased pulmonary fibrosis (Ask et al, 2008). Consequently, efforts were made to generate novel compounds that inhibit LPA generation (autotaxin inhibitors) and LPA receptors. The only phase 2 trials of an autotaxin inhibitor (ziritaxestat) in patients with IPF were canceled due to a lack of an effect (Maher et al, 2023). On the contrary, first-generation inhibitors of LPA1 have shown promising results in phase 2 trials (Palmer et al, 2018; Decato et al, 2022) where the decline in forced vital capacity (FVC), a key measurement of lung function, and collagen accumulation, as measured by extracellular matrix–neoepitope biomarkers, is statistically attenuated. One challenge for current LPA receptor antagonists is the aforementioned increase in LPA levels over time in patients with IPF. As demonstrated in Ca^2+^ assays and a vascular leakage assay, 2 competitive LPA1 antagonists in clinical development, HZN-825 and BMS-986278 (admilparant), can be readily washed out within 2 hours and displaced from LPA1, thereby reducing the effectiveness of the compounds (Birker-Robaczewska et al, 2025). Thus, as IPF progresses and LPA concentrations increase, the competitive antagonists may lose effect, as depicted in the upper panels of Fig. 1. Such effects may be able to be overcome in clinical practice with dosing, by increasing either the concentration as demonstrated by Birker-Robaczewska et al (2025) or frequency of drug administration. However, both approaches are not ideal as increasing concentrations lead to more adverse effects and increasing the frequency a drug must be taken reduces compliance. Similar data can be seen in the most recent phase 2 clinical data from the second-generation LPA1 antagonist admilparant, where 30 mg was completely ineffective, but 60 mg showed a decline in FVC after removing patients who had a dose reduction (Corte et al, 2025).

**Fig. 1:**
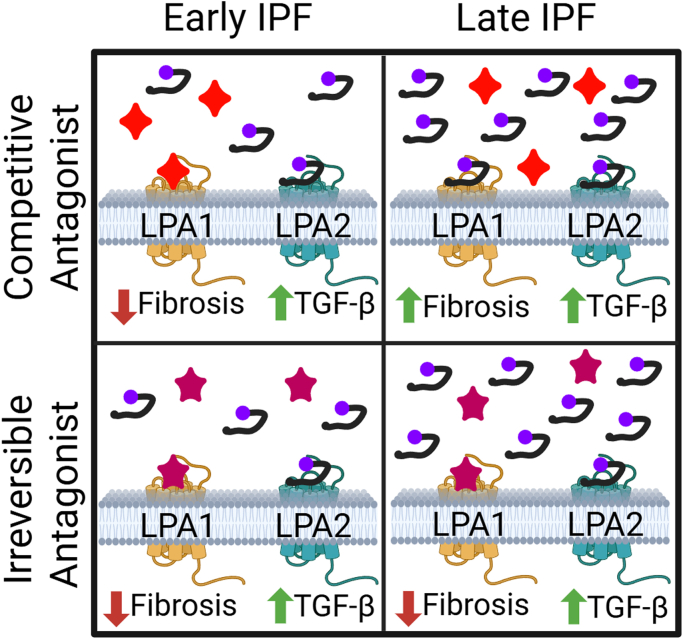
Competitive lysophosphatidic acid receptor (LPA)1 antagonists have a potential downside in treating idiopathic pulmonary fibrosis (IPF) compared with noncompetitive LPA1 antagonists. Because LPA concentrations and LPA1 receptors (not shown) increase as IPF progresses, competitive antagonists can be displaced from LPA1, thereby losing its antifibrotic effect. An irreversible antagonist may remain bound to LPA1 even in higher LPA concentrations, thereby retaining its antifibrotic effect. However, inhibiting LPA1 allows the LPA to bind to LPA2 and increase transforming growth factor (TGF)-*β* levels. The red 4-sided star represents a competitive LPA1 antagonist, whereas the mauve 5-sided star represents an irreversible LPA1 antagonist. LPA is depicted by a purple ball with a black tail. Created in BioRender by Andresen B (2025). https://BioRender.com/k50f949

Idorsia Pharmaceuticals have been generating piperidine-based compounds as LPA1 antagonists, leading to the development of ACT-1016-0707, an orally viable LPA1 antagonist (Lescop et al, 2024a,b). In a recent issue of *The Journal of Pharmacology and Experimental Therapeutics*, the Idorsia Pharmaceuticals group presents data demonstrating that ACT-1016-0707 has slow off-rate kinetics from LPA1 and the classical downward shift in concentration-response curves of an irreversible antagonist (Birker-Robaczewska et al, 2025). Unsurprisingly, ACT-1016-0707 remains effective for hours and consequently displays a significant reduction in the degree of bleomycin-induced IPF and many markers of fibrosis. However, ACT-1016-0707 did not statistically improve FVC in mice, similar to studies with nintedanib (Ackermann et al, 2017). Unfortunately, the comparisons between ACT-1016-0707, HZN-825, and admilparant were conducted only in cellular and vascular leakage experiments. Thus, it is unknown whether ACT-1016-0707 is superior to admilparant in a model of IPF. Such comparative data, specifically to admilparant, may be important when considering further developing ACT-1016-0707.

One shortcoming in the in vivo studies of ACT-1016-0707 is that animals were pretreated with ACT-1016-0707 1 day before the administration of bleomycin. Pretreatment is problematic because it does not model how patients are diagnosed with a disease and then treated with pharmaceuticals. The bleomycin-induced IPF model is rapid and reliable (Liu et al, 2017) but does not preclude treating mice at the onset of lung pathology (7 days after bleomycin treatment) as has been conducted with nintedanib (Ackermann et al, 2017). Studies conducted after lung function deteriorates are important as they mimic clinical situations. Moreover, because LPA levels increase in bleomycin-induced IPF (Tager et al, 2008), the bleomycin-induced IPF is an excellent model to test the hypothesis that ACT-1016-0707 is superior to a reversible antagonist, such as admilparant in the treatment of IPF.

Another consideration in developing LPA receptor inhibitors is the role of LPA2. Agonism of LPA1 and LPA3 does not lead to TGF-*β* activation, but activation of LPA2 leads to increased TGF-*β* (Xu et al, 2009). Therefore, a strong hypothesis can be generated regarding inhibiting both LPA1 and LPA2. When LPA levels increase in IPF, blocking LPA1, especially with an irreversible antagonist, theoretically leads to greater activation of LPA2. Because LPA2 leads to increased TGF-*β* (Xu et al, 2009), the expectation is that there would be continued pulmonary fibrosis and exacerbations of a patient’s IPF symptoms. The activation of LPA2 while blocking LPA1 with an irreversible antagonist like ACT-1016-0707 is modeled in the bottom panels of Fig. 1. Similar data are presented in human precision-cut lung slices where ACT-1016-0707 failed to normalize fibronectin and collagen deposition when treated with LPA and TGF-*β*1 (Birker-Robaczewska et al, 2025), demonstrating that TGF-*β* production can still lead to fibrosis when LPA1 is effectively inhibited. Furthermore, LPA signaling through LPA2 may explain why admilparant failed to normalize FVC (Corte et al, 2025). Antagonism of LPA1 and LPA2 should attenuate pulmonary fibrosis to a greater extent than an irreversible LPA1 antagonist due to the ability of LPA2 to increase TGF-*β* levels.

Despite the shortcomings and lack of direct comparison with admilparant in the study by Birker-Robaczewska et al (2025), the success of LPA1 antagonists in clinical trials supports continuing to develop ACT-1016-0707. Further steps should include additional species, and if ACT-1016-0707 remains safe and effectively reduces fibrosis across species, then clinical trials should begin. It will be interesting to compare phase 2 trials of ACT-1016-0707 with admilparant, as an irreversible antagonist may be able to be used at a lower concentration, and phase 2 studies with admilparant that failed to improve FVC at the lowest dose. It remains an open question whether using an irreversible antagonist of LPA1 is sufficient to halt IPF-mediated decline in FVC. Given the recent clinical data with admilparant, which were collected in the context of current antifibrotic therapy, most likely, due to the complex etiology of IPF, a combination of therapies is required to control IPF. The addition of LPA1 antagonists to the current therapeutic options will likely significantly improve the treatment of IPF; however, it is equally likely that additional novel targets will be required to stabilize FVC in IPF patients.

## Conflict of interest

The authors declare no conflicts of interest.
